# Negative Pressure Smart Patch to Sense and Heal the Wound

**DOI:** 10.1002/advs.202408077

**Published:** 2024-11-28

**Authors:** Xing Liu, Peng Zhao, Xiaozhuo Wu, Yawei Zhao, Feifan Zhou, Ying Luo, Xiaoli Jia, Wen Zhong, Malcolm Xing, Guozhong Lyu

**Affiliations:** ^1^ Engineering Research Center of the Ministry of Education for Wound Repair Technology Jiangnan University Affiliated Hospital of Jiangnan University Wuxi 214000 China; ^2^ Wuxi School of Medicine Jiangnan University Wuxi 214000 China; ^3^ Department of Mechanical Engineering University of Manitoba Winnipeg R3T 2N2 Canada; ^4^ Department of Biosystems Engineering University of Manitoba Winnipeg R3T 2N2 Canada

**Keywords:** drug delivery, glucose‐sensing, microspheres, pH‐sensing, wound monitoring

## Abstract

Negative pressure wound therapy (NPWT) offers significant advantages in terms of rate and time for healing through generating sub‐vacuum to draw out inflammatory exudate and promote wound closure. However, continuous drainage probably leads to healing delay due to the lack of information about the real status of the wound bed and the potential risk of infection. To address this concern, printed Negative Pressure Smart Patch (NPSP) is reported by integrating smart real‐time sensing acidity (infection) and glucose, and anti‐infection into NPWT systems. In addition, NPSP delivers vancomycin through chitosan porous microspheres under negative pressure to modulate wound healing. Compared with NPWT, NPSP projects a promising approach to removing bacteria, reducing local inflammation, and accelerating healing in a short period of time.

## Graphical abstract

Smart real‐time pH and glucose sensors, along with anti‐infective properties, are integrated into a negative pressure wound therapy system to continuously monitor the wound microenvironment and eliminate bacteria without requiring dressing changes.

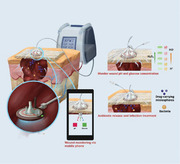

## 1 Introduction

The skin is an effective barrier in terms of protecting the organism from bacteria, foreign bodies, and dehydration.^[^ ^1^ ^]^ Once skin damage occurs, the body activates a series of procedures to protect the wound and inner organs. Wound healing is a complex multistep scenario involving overlapping phases of hemostasis, inflammation, proliferation, and remodeling.^[^ ^2^, ^3^ ^]^ There is growing evidence that the transition from the inflammatory to the proliferative phase is a critical step in the healing process and that if inflammation persists over time, it may lead to tissue damage and ultimately to scarring or chronic wounds.^[^ ^4^ ^]^

These skin‐related diseases have caused a growing health and economic burden on our society.^[^ ^5^, ^6^ ^]^ To date, a variety of dressings have been designed to promote wound healing, however, despite the variety of clinical options available, existing wound dressings still fail to meet certain specific needs.^[^ ^7^, ^8^, ^9^, ^10^, ^11^ ^]^ Negative pressure wound therapy (NPWT) is the most common and versatile technology that significantly accelerates wound healing.^[^ ^12^ ^]^ Generally, NPWT consists of a vacuum source, a sealing membrane, a negative pressure patch, and a negative pressure sponge, enabling the creation of continuous or intermittent negative pressure in the wound/trauma area.^[^ ^13^ ^]^ NPWT aids in reducing peri‐lesion edema, maintaining an optimal moist wound environment, eliminating infected fluid and exudate, enhancing blood flow, and promoting angiogenesis.^[^ ^14^, ^15^, ^16^ ^]^ Widely recommended for various acute and chronic wounds, NPWT accelerates the healing process of pressure wounds, diabetic leg ulcers, burns, and other injuries.^[^ ^13^, ^17^, ^18^, ^19^, ^20^, ^21^ ^]^ Although numerous clinical studies have demonstrated the significant effects of NPWT in improving wound healing rates and shortening healing times, the technique faces certain bottlenecks in controlling infection and monitoring wounds.^[^ ^22^, ^23^, ^24^ ^]^ First, necrotic tissue and sponge dressings in the wound during NPWT may promote bacterial growth and spread of infection. Second, currently, visual inspection of the wound is mainly relied upon to assess the status of the wound, but NPWT hinders this visual assessment by completely covering the wound surface, which hinders healthcare professionals from achieving accurate diagnosis and timely decision‐making. They mainly monitor the wound condition by repeatedly changing the dressing, a practice that is not only costly, time‐consuming, and painful for patients, but may also endanger their wound recovery and even their lives due to delayed treatment.^[^ ^25^, ^26^ ^]^

The field of intelligent wearable medical devices (IWMDs) is experiencing rapid advancements, particularly in the realms of biosensing and drug delivery for disease identification.^[^ ^27^, ^28^, ^29^, ^30^ ^]^ Notably, the integration of optical sensors with smartphones facilitates a convenient, economical, and user‐friendly approach to on‐site detection, eliminating the need for specialized expertise.^[^ ^31^, ^32^ ^]^ Concurrently, wearable drug delivery devices (WDDs) are increasingly supplanting conventional methods of medication administration.^[^ ^33^, ^34^, ^35^ ^]^ In the present moment, researchers are diligently endeavoring to amalgamate biosensors with WDDs, with the aim of developing a cohesive system. Such an integrated system would not only recognize physiological or pathological indicators, but would also be able to administer therapeutic drugs, manage drug dosages with superior accuracy, avoid systemic side effects, and improve personal healthcare beyond the limitations of traditional treatment modalities.^[^ ^36^, ^37^ ^]^ Therefore, it is particularly crucial to develop a new NPWT system that can monitor the wound status in real‐time without changing the dressing and remove bacteria effectively. Thus, we consider 3D printing technology for this since it has been increasingly used in medical devices to provide personalized medical solutions by customizing the size, shape, and structure according to the specific needs and anatomical features of the patient.^[^ ^38^, ^39^, ^40^ ^]^

Meanwhile, we also consider pH and glucose sensing for NPWT. pH is a key indicator for detecting the status of the wound and can indicate the current state of the wound.^[^ ^41^, ^42^, ^43^ ^]^ Another wound marker is glucose concentration, which has been found to correlate with healing progress.^[^ ^44^ ^]^ Acute wounds have high glucose concentrations, and their monitoring should be crucial for healing, especially for diabetic patients.^[^ ^45^, ^46^ ^]^

In light of these considerations, we integrated smart sensors into NPWT systems for a Negative Pressure Smart Patch (NPSP) using 3D printing. The NPSP utilizes hydrogels containing glucose oxidase (GOx) and horseradish peroxidase (HRP), as well as injectable microspheres containing phenol red, to monitor pH and glucose levels within the wound during negative pressure therapy. Additionally, the NPSP integrates diagnostics and therapeutics in the same smart patch, enabling local delivery of microspheres based on the wound microenvironment. We first conducted in vitro experiments to validate the NPSP's ability to monitor pH and glucose concentration and developed fitting equations based on RGB images of the NPSP with these parameters. Next, we verified the antimicrobial effect of drug‐loaded microspheres in vitro. Finally, in* in vivo* experiments, we further validated the monitoring function and drug delivery effect of the NPSP.

## 2 Results and Discussion (NPSP System Materials, Design, Integrated Architecture and Performance Characteristics)

### 2.1 Constituent Materials, Design Layout, and Manufacturing Procedure

**Figure 1:**
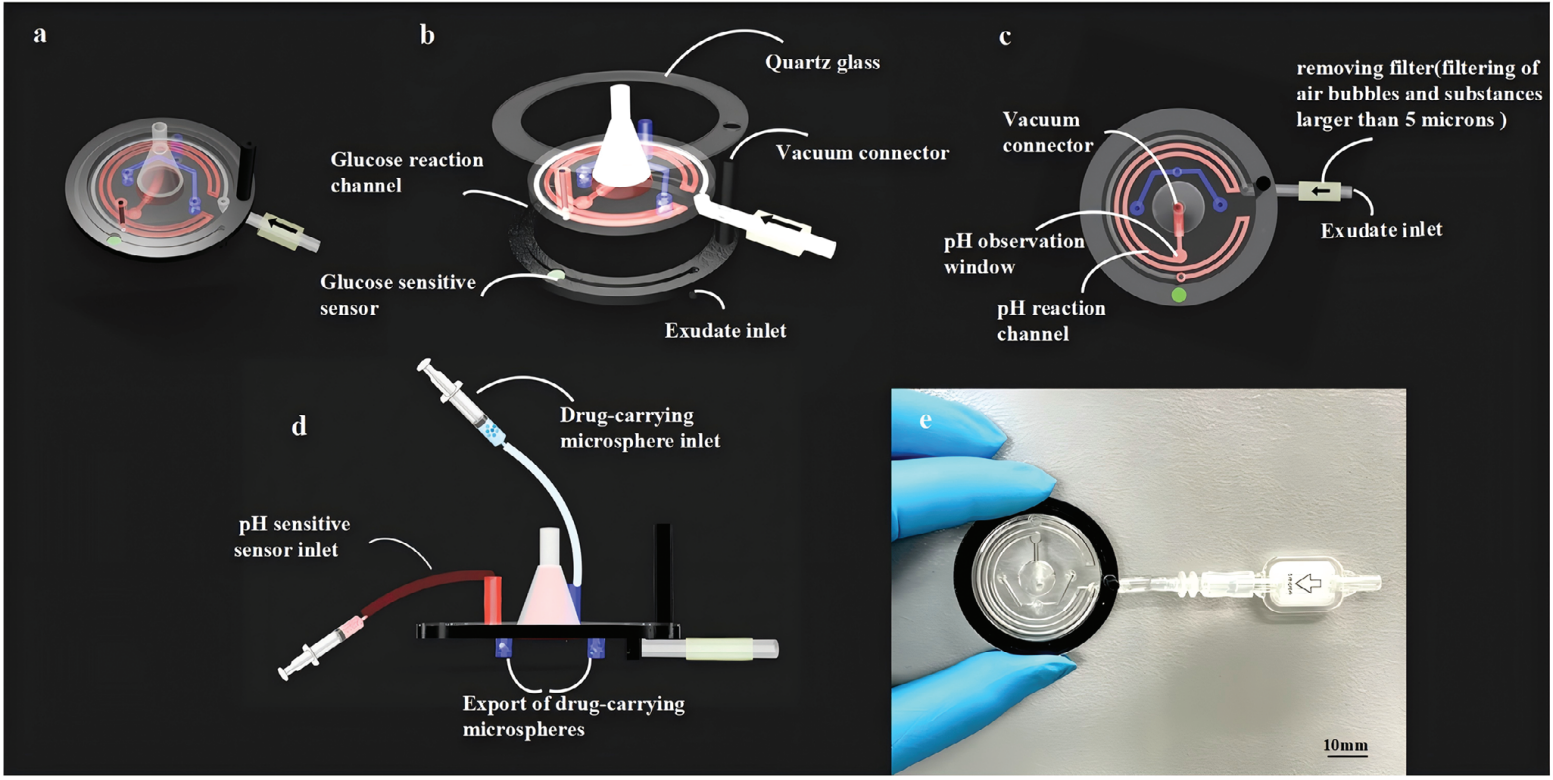
3D and physical drawings of the NPSP. a) Overall view of the NPSP; b, c) Structural view of the NPSP; d) Side view of the NPSP; e) Physical view of the NPSP.

The NPSP system consists of six core components: a transparent epoxy channel, a black epoxy channel, a quartz glass cover, a removable filter, a glucose sensor, and a pH sensor (**Figure** **1**a; Video S1, Supporting Information). In the glucose channel (Figure 1b), to better observe the fluorescence intensity of the glucose sensor under UV light, we printed the glucose reaction channels using black epoxy resin and encapsulated them in quartz glass with transparency and transmission ratio.^[^ ^47^ ^]^ To effectively observe the color change of the pH sensor in the channel, transparent epoxy resin was used. This epoxy channel is designed for the integration of pH reaction, drug microsphere delivery, and exudate drainage (Figure 1c). Additionally, this NPSP system addresses issues such as dye leaching and non‐repeatability encountered in luminescence systems. When the NPWT system is connected to a vacuum source, the air within the negative pressure sponge is evacuated, causing its thickness to swiftly be reduced from 12 to 5 mm. To avoid overloading the wound and ensure smooth delivery of the drug‐carrying microspheres, the outlet for the drug‐carrying microspheres is designed as a 3 mm long channel (Figure 1d). Figure 1e shows the NPSP device.

**Figure 2:**
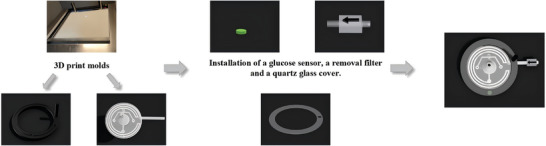
Fabrication process and installation of NPSP.

**Figure** **2** illustrates the manufacturing process. In contrast to commercial negative pressure patches, the NPSP contains microfluidic channels for monitoring the wound environment (Figure S1a, Supporting Information). Figure S1b (Supporting Information) depicts the liquid state in the pH sensor, with and without a filter. The filter eliminates air bubbles to maintain air bubble‐free liquid in the NPSP microfluidic channel. Figure S1c (Supporting Information) demonstrates the assembly diagram of the NPSP and the negative pressure sponge.

### 2.2 In Vitro Glucose Monitoring and Smartphone Sensing

**Figure 3:**
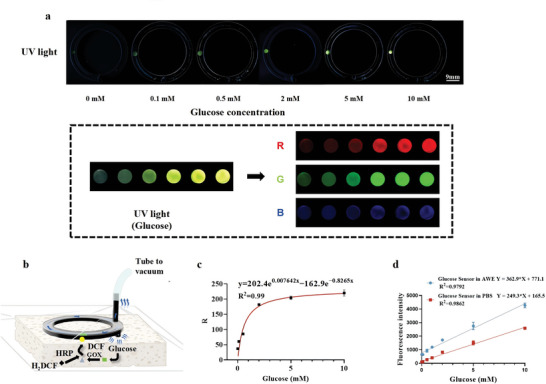
Images for in vitro monitoring of glucose concentration. a) Photographs and RGB images of the glucose sensor at different glucose concentrations; b) Scheme of the glucose sensor for detecting glucose concentration in wound exudate; c) Corresponding fitted curves for glucose concentration (*n* = 3); d) Linearity of fluorescence intensity and glucose concentration in glucose sensor (*n* = 3).

Alginate microspheres containing GOx and HRP were pressed into a mold to make a glucose sensor, which was subsequently placed into the glucose‐responsive channel of the NPSP device, and 10 µl of 0.5 × 10^−3^ m 2',7'‐Dichlorodihydrofluorescein diacetate (H_2_DCF) substrate solution was added dropwise (Figure S2a, Supporting Information). After the hydrogel dried slightly, the channel was encapsulated and negative pressure suction was performed. Figure S2b (Supporting Information) illustrates the catalytic reactions mediated by GOx and HRP, which are central to the sensor's operation. The working principle of the glucose‐sensitive sensor in NPSP is shown in **Figure** **3**b. As shown in Figure 3a, the response of the glucose sensor was visualized under 365 nm UV irradiation to capture the glucose‐impregnated sensor and its RGB image. In addition, the optical path diagram shown in Figure S2C (Supporting Information) illustrates the sensor's operating and capturing environment. In this study, the NPSP demonstrated a glucose detection range from 0.1 to 10 × 10^−3^ m. An incremental increase in glucose concentration corresponded to a heightened red signal from the glucose sensor, establishing a functional correlation between glucose levels and the R‐value (Figure 3c). This relationship was utilized to calculate wound exudate glucose concentrations by fitting the curve logarithmically. We extracted the sensitivity from the fitted curve, which was determined to be 0.115 m ^−1^ at a glucose concentration of 0.2 × 10^−3^ m. Notably, as the glucose concentration increases, the sensitivity decreases.

A linear positive correlation between glucose concentration and the fluorescence intensity of dichlorofluorescein (DCF) when catalyzing the oxidation of glucose in PBS and artificial wound exudate (AWE) solutions indicates that the glucose sensor maintains its function and is not adversely affected by proteins and other substances commonly found in osmotic solutions (Figure 3d). In addition, we explored the effect of red blood cells on the performance of the glucose sensor (Figure S2d, Supporting Information). At a 40% red blood cells concentration, the glucose sensor's fluorescence intensity showed a notable drop compared to the control, indicating substantial interference from high red blood cells levels. However, at concentrations below 20%, red blood cells had little impact on sensor performance. Thus, glucose sensors are suitable for monitoring glucose in biological settings, but caution is needed regarding red blood cells interference, particularly in wound care where blood presence is high.

### 2.3 In Vitro pH Monitoring and Smartphone Sensing

pH sensors of phenol‐loaded alginate microspheres were loaded into a syringe, which was then connected to the channel of the NPSP. Once negative pressure was produced, the pH microsphere sensors were sucked into the pH channel of the device.

**Figure 4:**
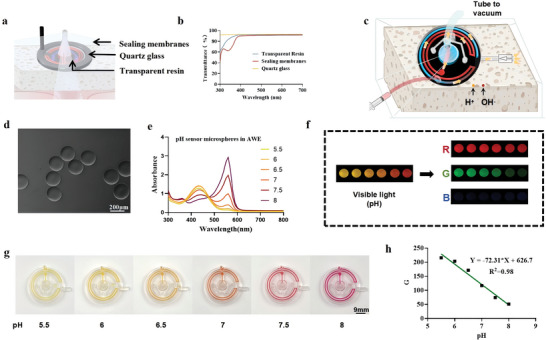
Images for in vitro pH monitoring. a) Position of the transparent resin, sealing film, and quartz glass during operation of the NPSP; b) Transmittance of the transparent resin, sealing film, and quartz glass cover sheet; c) Scheme of operation of the pH‐sensitive sensor; d) Microscopic image of the pH ‐sensitive hydrogels; e) UV‐visible absorption spectra of the pH‐sensitive hydrogels at different pH; f) RGB images of the hydrogel at different pH; g) Images of the pH‐sensitive sensor at different pH; h) Plot of the linear regression equation of G‐value on pH (*n* = 3).

**Figure** **4**a depicts the schematic of the NPSP in NPWT. The pH sensor is encased in resin and overlaid with a negative pressure film, while the glucose sensor is ensconced beneath quartz glass, similarly accompanied by a negative pressure film. Figure 4b shows that the sensor cover has very high transmittance in the 300–700 nm wavelength range, suggesting that the cover material does not obstruct the visibility of sensor color changes. Figure 4c is a schematic diagram of how the pH‐sensitive sensor works, with the arrows representing the flow path of the reaction solution. Figure 4d is a microscopic image of the pH microsphere sensors. Generally, skin tissue typically has a pH range of 4 to 6, while wound tissue infected with bacteria may have a pH range of 7 to 8. However, as the degree of infection decreases, the pH also decreases.^[^ ^48^, ^49^, ^50^ ^]^ NPSP indicates a clear color change visible in the pH range (5.5–8) relevant to the wound healing process (Videos S2, S3, Supporting Information). As shown in Figure 4e, the adsorption peak of the pH microsphere sensors at 560 nm corresponds to the n–π* jump from benzene exciton to quinone exciton as the surrounding solution becomes more alkaline.^[^ ^51^ ^]^ Figure 4f,g display photographs of the pH‐sensitive sensor at different pH levels. As can be seen, the color of pH microsphere sensors changed from yellow to red with the increase of pH, while the green signal of pH microsphere sensors hydrogel decreased. Ultimately, a final linear regression equation of G‐value on pH was determined. Therefore, the pH value of the wound exudate can be derived by calculating according to the equation (Figure 4h). From the equation, we extracted a sensitivity value of −72.31, which is negative, indicating an inverse relationship between pH and G‐value. In other words, as the pH increases, the G‐value decreases. Furthermore, the scanning spectra and absorption values of the pH sensor microspheres tested in AWE were comparable to those observed in the PBS solution, suggesting that substances such as proteins in AWE do not interfere with the results (Figure S3a,b, Supporting Information).

pH monitoring can be conducted through two distinct methods: colorimetric and electrochemical approaches.^[^ ^7^ ^]^ Optical methods provide the advantages of simplicity and low cost as compared to electrical methods, enabling the conversion of physiological information in the wound into a light signal for direct observation. Numerous indicators and dyes exhibit color changes under varying pH conditions, including bromocresol violet, phenol red, and naphthol phthalein.

This study employed a microfluidic channel integrated with microspheres loaded with phenol red to overcome the limitations of optical sensors that are susceptible to dye leakage and lack infinite reproducibility. Additionally, the NPSP demonstrated the capability to monitor glucose concentration; however, glucose detection was found to be non‐reproducible. We developed a comparison table to assess the performance of flexible wearable sensors for optical pH detection, aiding in the evaluation of our biosensors’ efficacy for pH monitoring (**Table** **1**).^[^ ^52^, ^53^, ^54^, ^55^, ^56^ ^]^

**Table 1:** Performance comparison of flexible wearable sensors for optical pH detection.

Method	pH range	Reusability	Leaching or not	Feature	References
Colorimetry	4–8	No	Yes	Portable and quantitative	[52]
Fluorescence	5–9	No	Yes	Portable, quantitative, real‐time, and remote	[53]
Colorimetry	5–8	No	No	Low cost and convenience	[54]
Colorimetry	5–8	No	Yes	Simple operation, easy detection, fast response, and high sensitivity	[55]
Fluorescence	5.5–7.5	No	Yes	Portable and semi‐quantitative	[56]
Colorimetry	5.5–8	Yes	No	Reusable, low cost, no dye leakage	Current work

### 2.4 Preparation and Characterization of Drug**‐**Loaded Microspheres, pH Sensors, and Glucose Sensors

Chitosan microspheres (CSMs) were successfully prepared by high‐pressure electrospray method using 1% chitosan solution. Subsequently, different concentrations of vancomycin were loaded onto the CSMs by simple adsorption (labeled as V/CSM1, V/CSM2, V/CSM3, and V/CSM4). The FTIR spectra of CSM, vancomycin, and V/CSM (Figure S4, Supporting Information) show specific peaks: CSM features N─H/O─H stretching at 3407 cm^−1^, C─H stretching at 2958 cm^−1^, N─H bending at 1587 cm^−1^, C─N stretching at 1377 cm^−1^, and C─H bending at 1064 cm^−1^. Vancomycin features N─H/O─H stretching at 3290 cm^−1^, C─H stretching at 2958 cm^−1^, C═O stretching at 1649 cm^−1^ (amide I), N─H bending at 1587 cm^−1^ (amide II), benzene ring vibrations at 1494 cm^−1^, ─CH₃ bending at 1395 cm^−1^, and C─N stretching at 1310 cm^−1^ (amide III). The V/CSM s' spectrum combines both, with reduced drug peak intensities post‐encapsulation, indicating successful drug loading.

**Figure 5:**
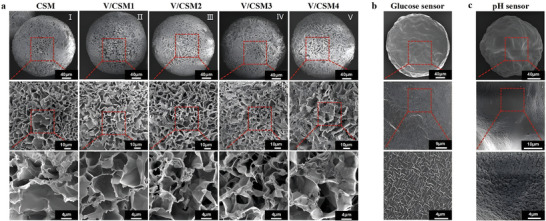
SEM images of microspheres and sensors. a) SEM photographs showing the morphology of various microspheres prepared in this study; b) SEM photographs of Glucose Sensor; c) SEM photographs of pH Sensor.

The scanning electron microscopy (SEM) images vividly illustrate the distinctive porous structure of the CSM surface when compared to the hydrogel sensor (**Figure** **5**a–c). This porous configuration not only amplifies the overall surface area available for drug molecule adsorption, but also offers ample storage room for drugs. There was no significant morphological difference between the drug‐loaded and the original microspheres, and all the microspheres remained spherical with the same average particle size. But the voids of the vancomycin‐loaded microspheres were encapsulated by vancomycin powder, which was different from the unloaded vancomycin CSMs. The quantity of powder within the pores increased with the concentration of the vancomycin solution. Given that the pore dimensions of negative pressure sponge typically range between 100 and 250 µm, microspheres with diameters primarily falling within the 280–300 µm range were fabricated to ensure effective drug transport while preventing their entrapment within the sponge pores under negative pressure.

### 2.5 Antibiotic Diffusion (Zone of Inhibition), Antimicrobial Activity Test, and In Vitro Release of Vancomycin

**Figure 6:**
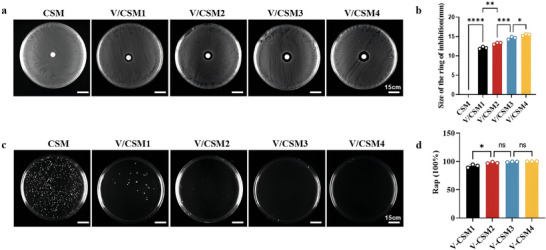
Diffusion and release of vancomycin from microspheres. a) Vancomycin spreads from the central hydrogel disc, killing surrounding bacteria and leaving a clear zone on each plate; b) Quantitative analysis of ZOI of *S. aureus* by V/CSM and CSM (control); c) *S. aureus* suspensions were enumerated using the plate spreading method after four hours of co‐culture with different microspheres; d) Quantitative analysis of bacteria in suspensions. Rap: Ratio of planktonic bacteria. Results for insets (b) and (d) are expressed as mean ± standard deviation, *p < 0.05, **p < 0.01, ***p < 0.001, ****p < 0.0001. Statistical analyses were performed using one‐way ANOVA followed by Tukey post‐hoc test, *n* = 3.

Most wound infections are caused by *Staphylococcus aureus* (*S. aureus*).^[^ ^57^ ^]^ In the present work, the bactericidal activity of vancomycin released from CSM microspheres was determined using a zone of inhibition (ZOI) assay. As the CSM microspheres did not contain vancomycin, no inhibition zone was formed against *S. aureus*. However, V/CSM1, V/CSM2, V/CSM3, and V/CSM4, which contained different concentrations of vancomycin, formed distinct inhibition zones on *S. aureus* lawns grown on agar plates (**Figure** **6**a). The data indicates a clear correlation between ZOI and vancomycin concentration, which is in line with prior research (Figure 6b).^[^ ^58^ ^]^

Strikingly, while a large number of colonies belonging to the CSM group grew on agar plates, whereas obviously fewer colonies belonging to the V/CSM1, V/CSM2, V/CSM3, and V/CSM4 groups were observed (Figure 6c). The Rap against planktonic bacteria for V/CSM1, V/CSM2, V/CSM3, and V/CSM4 was 95.26% ± 0.32%, 98.64% ± 0.68%, 99.61% ± 0.42% and 99.73% ± 0.31%, respectively. CSM microspheres loaded with vancomycin exhibited higher anti‐planktonic bacteria rates with increasing vancomycin concentrations. However, the Rap was not statistically significant (*P *> 0.05) when the vancomycin concentration exceeded 2 mg mL^−1^ (Figure 6d). Therefore, we do not need to use excessive amounts of vancomycin to clear the bacteria or there will be cytotoxicity.^[^ ^59^ ^]^ At the same time, these results also determine the dose to be administered in future in vivo experiments.

Diabetic ulcers are very susceptible to infection, and they require urgent treatment once infected. If not treated quickly, severe infections may lead to necrosis and threaten patient's' life.^[^ ^60^, ^61^ ^]^ Therefore, after the NPSP detects that the wound is infected during NPWT, drug‐carrying microspheres are rapidly delivered through the drug delivery channel. In this study, chitosan microspheres are used to release vancomycin, which is essential for early control of infection. The loading of vancomycin in microspheres was measured with the help of UV spectrophotometer to determine the amount of vancomycin released. CSM microspheres (V/CSM1, V/CSM2, V/CSM3, V/CSM4) were prepared with different concentrations of vancomycin solutions with vancomycin contents of 146.41 ± 5.11, 162.13 ± 7.78, 172.94 ± 5.25, and 187.58 ± 2.99 µg mg^−1^, respectively. Notably, the concentration of vancomycin was linearly related to the absorbance at 281 nm. Therefore, the concentration of vancomycin can be estimated from its optical density (OD) value. Based on Figure S5 (Supporting Information), at 20 min, the release rates of vancomycin from V/CSM1, V/CSM2, V/CSM3, and V/CSM4 were 85.2% ± 1.2%, 85.28% ± 4.7%, 91.2% ± 1.2%, and 91.25% ± 2.3%. The drug‐loaded microspheres rapidly released vancomycin into acutely contaminated wounds, creating an environment that is relatively sterile, which is important for timely control of infection.

### 2.6 Biocompatibility

**Figure 7:**
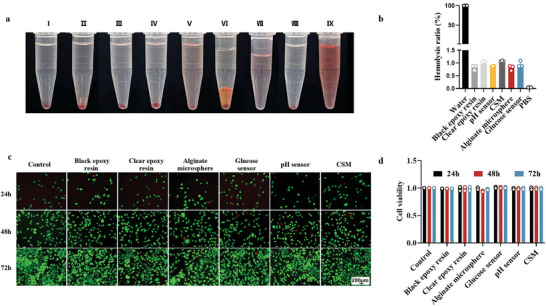
In vitro experiments on biocompatibility and cytotoxicity. a) Captions were representative images of hemolysis evaluation. I–IX represent Black Epoxy Resin, Clear Epoxy Resin, Alginate microsphere, Glucose sensor, pH sensor, CSM, pH sensor added to PBS without blood cells as background, negative control, and positive control, respectively; b) Rate of hemolysis; c) Live/dead cell staining images; d) Measurement of cell viability by CCK8 assay. For insets (b) and (d), data are presented as mean ± standard deviation, *n = 3*.

The hemolysis test is a well‐established method for assessing the hemocompatibility of biomaterials in vitro.^[^ ^62^ ^]^ The proportion of hemolyzed red blood cells for the above types of materials was almost the same as that of the negative control group (**Figure** **7**a). As shown in Figure 7b, quantitative analysis of hemolysis rate indicated that all materials had a hemolysis rate of less than 1%, which met the rate limits standardized by ASTM F756 (American Society for Testing and Materials).

Furthermore, cytocompatibility is a crucial indicator of the in vitro toxicity of biomaterials. Similar to cells of the control group, cells cultured with NPSP subassembly extracts also maintained good cell morphology and viability (Figure 7c,d).

### 2.7 In Vivo Studies

#### 2.7.1 Antimicrobial Properties of Negative Pressure Smart Patch Administration

**Figure 8:**
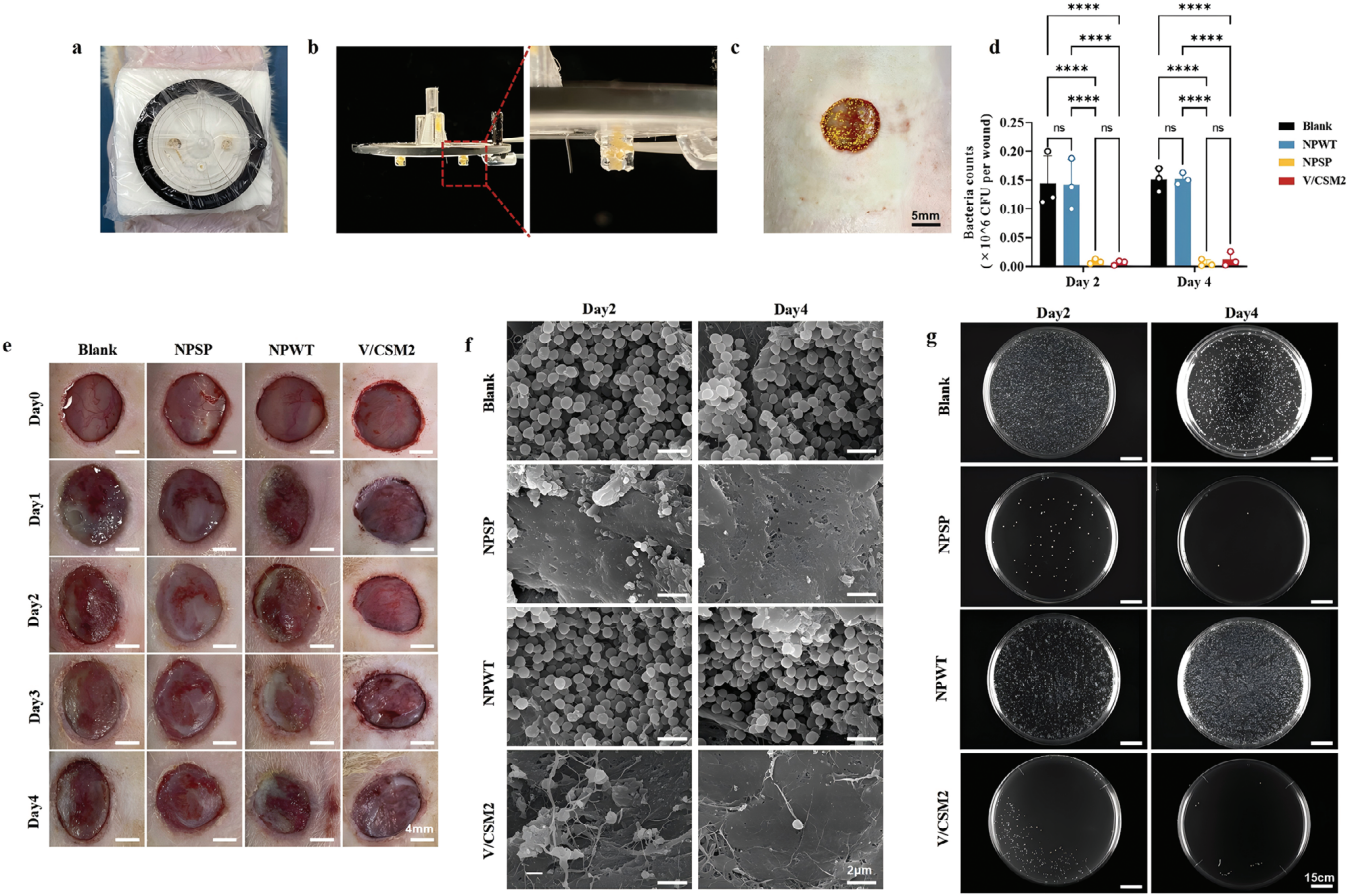
Negative pressure drug delivery via NPSP. a) Photograph of the NPSP on a rat; b) Photographs of the NPSP after the delivery of drug microspheres via the device; c) Photograph of the surface of the rats' wounds at after drug administration; d) colony count analysis of infected tissues after different treatments; e) Images of the wounds in different treatment groups; f) SEM examination of the surface of the wound bed infected with *S. aureus*; g) Images of the bacterial colonization of infected tissues after different treatments. Results for inset d are expressed as mean ± standard deviation, ****p < 0.0001. Statistical analyses were performed using one‐way ANOVA followed by Tukey post‐hoc test, *n* = 3.

V/CSM2 showed significant antimicrobial activity. To validate the effect of negative pressure drug delivery in vivo, we created full‐thickness circular wounds of 1 cm in diameter on the back of rats and set up four groups for testing: 1) an untreated infected group; 2) an NPWT group; 3) an NPSP drug delivery group (NPSP group); and 4) a group treated by drug‐carrying microspheres alone (V/CSM2 group). This design was aimed at evaluating the effect of drug microspheres delivered via NPSP on the local wound infection. In the study, we successfully delivered drug‐loaded microspheres to the trauma site via NPSP (**Figure** **8**a–c; Video S4, Supporting Information).

After determining that V/CSM2 had been successfully delivered to the wound, its bactericidal effect was further investigated. Figure 8e shows photographs of the wounds in different treatment groups. With regard to the SEM results, the results were similar in the open infection group and the NPWT group, with *S. aureus* aggregating and multiplying, gradually forming grape‐like clusters on the surface. However, in the NPSP and V/CSM2 groups, *S. aureus* colonies were found to be sparsely distributed on the tissue surface on day 2 after treatment. By day 4 after treatment, there was almost no detectable *S. aureus* distribution on the tissue surface (Figure 8f). This significant reduction in bacterial presence highlights the efficacy of the treatments in controlling infection at the wound site.

To further compare bactericidal efficacy between groups, *S. aureus* in tissue was quantified to obtain the number of bacteria in the wound at each time point. The antimicrobial efficacy of the NPWT group, NPSP group，and V/CSM2 group were assessed using colony plate counts (Figure 8d,g). Previous studies have verified that NPWT has some bactericidal ability.^[^ ^63^ ^]^ Nevertheless, the colony forming unit counts of the blank infected tissues in this experiment were very similar to those of the NPWT group, suggesting that NPWT had little or no bactericidal effect when used in infected wounds. This may stem from the short duration of NPWT operation in this experiment. In contrast, both the NPSP group and the V/CSM2 group exhibited strong bactericidal effects against *S. aureus* at the infected site, with no significant difference between the two groups.

**Figure 9:**
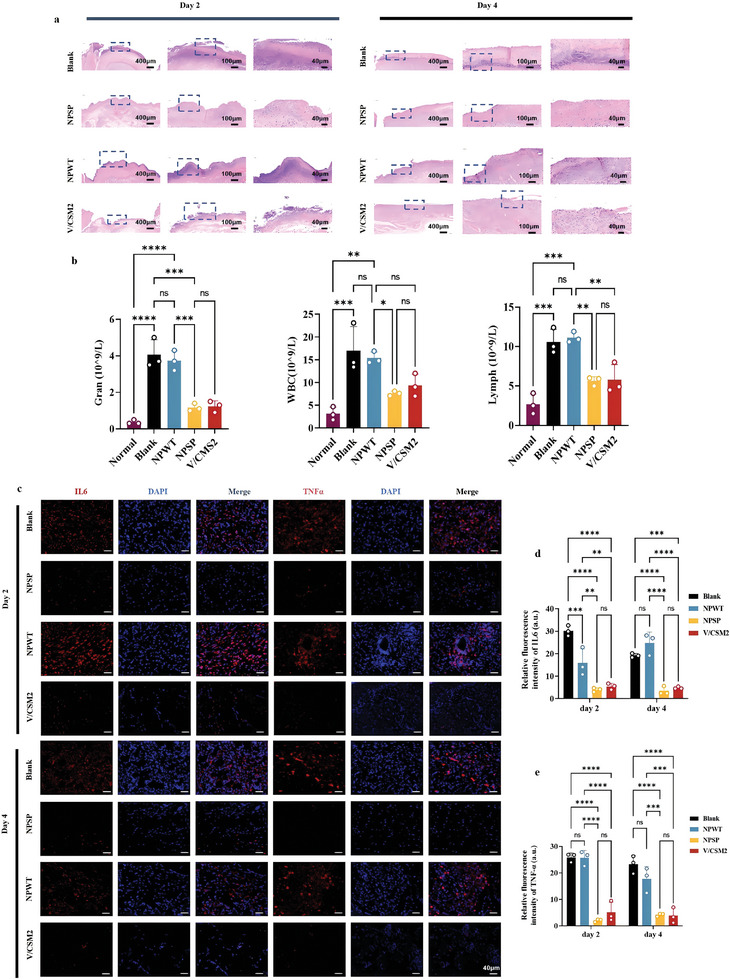
Negative pressure drug delivery through NPSP reduces the inflammatory response of infected wounds. a) H&E‐stained images of wound tissues on days 2 and 4 after different treatments; b) Blood biochemical indices of rats; c) Representative images of IL‐6 and TNF‐α immunostaining in wound tissues on days 2 and 4 after different treatments; d,e) Cytokine levels were measured in wound tissues on days 2 and 4 after different treatments. Results for insets b, d and e are expressed as mean ± standard deviation, *p < 0.05, **p < 0.01, ***p < 0.001, ****p < 0.0001. Statistical analyses were performed using one‐way ANOVA followed by Tukey post‐hoc test, *n* = 3.

Hematoxylin and eosin (H&E) staining was conducted on days 2 and 4 after treatment to assess bacterial invasion and inflammation in different wounds. Normally, once wound tissue is infected by exogenous bacteria, inflammatory cells migrate rapidly to the infection site.^[^ ^64^ ^]^ Therefore, as can be observed from a representative image of H&E staining (**Figure** **9**a), a large number of inflammatory cells were visible in the superficial layer of the wounds in both the blank and NPWT groups, with exudation of fibrin fluid. In contrast, only a small amount of inflammatory cell infiltration was present in the NPSP and V/CSM2 groups, indicating good debridement. As shown in Figure 9b, after 4 days of treatment, blood biochemical indicators including concentrations of white blood cells (WBC), neutrophils (Gran), and lymphocytes (Lymph) were obviously decreased in the NPSP and V/CSM2 groups as compared with the control and NPWT groups. This phenomenon implied that *S. aureus* infections were effectively suppressed.

We further examined the immunofluorescence staining of TNF‐α and IL‐6 in tissues with different treatments. As shown in Figure 9c, a large number of red fluorescent dots were observed in the wounds of both the NPWT group and the blank group, indicating high levels of IL‐6 and TNF‐α and an obvious inflammatory response. However, the fluorescence intensity was significantly lower in the NPSP group and the V/CSM2 group, indicating that the use of NPSP in the early stages of an infected wound helps to reduce inflammation (Figure 9d,e).

In conclusion, the results indicate that the NPSP and V/CSM2 groups exhibited similar bacterial clearance capabilities, suggesting that NPSP's ability to deliver drug‐carrying microspheres to the wound surface under negative pressure conditions is as effective as direct drug delivery. Furthermore, this finding paves the way for future therapeutic strategies that combine negative pressure with drug delivery, allowing these two modalities to work in synergy to enhance wound healing and improve infection control.

#### 2.7.2 NPSP In Vivo Detection of Glucose and pH Concentration

**Figure 10:**
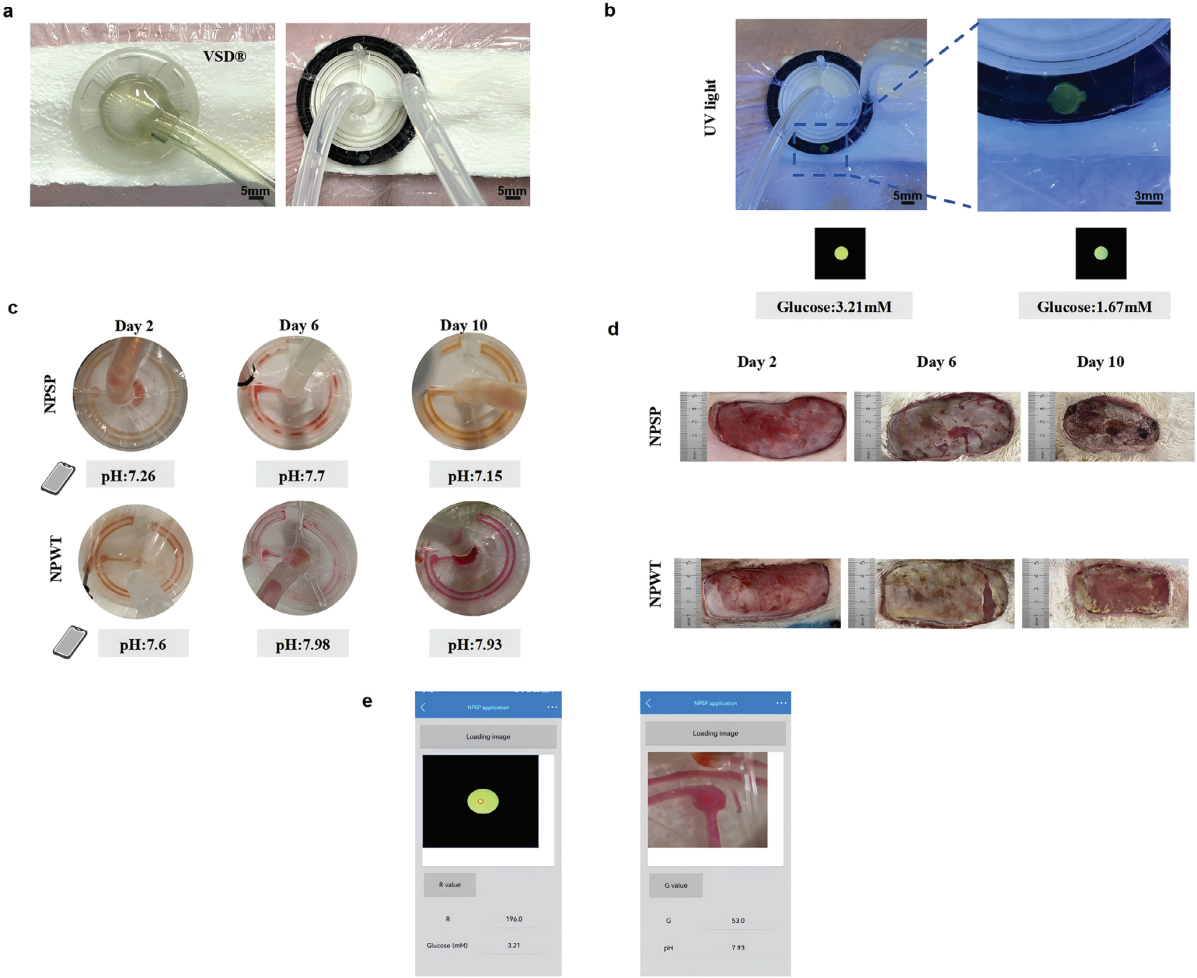
NPSP in vivo monitoring of pH and glucose concentrations. a) Photographs of an NPSP applied to a rabbit model (right) as compared to a conventional negative pressure patch (left); b) Photograph of an NPSP detecting exudate glucose concentration in a rabbit model; c) The NPSP responds to changes in pH associated with bacterial infection in wounds treated with NPSP and NPWT; d) Photographs depicting the progression of infected wounds under varying treatment conditions over time; e) Schematic diagram of the smartphone application to recognize pH and glucose concentration.

In vitro assessments demonstrated that the NPSP effectively monitors both pH and glucose levels within wound environments. NPWT is widely employed for managing exudate, to further assess the NPSP’s capacity to monitor pH and glucose concentrations amidst significant exudate volumes, we utilized this system in New Zealand White rabbit wound models. For experimental purposes, we excised a 30 cm^2^ wound on the dorsum of a New Zealand White rabbit to conduct two distinct treatment trials: 1) NPWT on infected wounds, and 2) NPSP on infected wounds (**Figure** **10**a). The NPSP displayed varying fluorescence intensities corresponding to the glucose concentrations in wounds, indicating that the exudates could interact with glucose sensors via glucose‐responsive channels (Figure 10b; Video S5, Supporting Information).

Normally, healthy skin has a pH between 4 and 6, which is slightly acidic and prevents bacteria from multiplying.^[^ ^65^, ^66^ ^]^ However, once a wound becomes infected, the pH changes to alkaline, mainly due to the presence of bacteria.^[^ ^67^, ^68^ ^]^ Then, as wound healing progresses, the pH begins to gradually decrease.^[^ ^69^ ^]^ Subsequent monitoring of wound pH with the NPSP revealed a color change in the sensor to red on day 6 after treatment, signaling an increase in wound pH. As shown in Figure 10c, by day 10, the pH levels in the NPSP group diminished, whereas those in the NPWT group remained relatively stable (Videos S6, S7, Supporting Information). Those results suggest enhanced wound healing and reduced bacterial proliferation in the NPSP group. The behavior of pH changes observed by NPSP is consistent with pH probe measurements. Figure 10d demonstrates macroscopic conditions of the wound under different treatments.

To enable wireless monitoring of wound infection and exudate glucose levels, we developed a smartphone application called the “NPSP application”, which rapidly identifies wound pH and glucose concentration using mobile phone imaging. Figure 10e illustrates the application interface for a smartphone application to recognize wound pH and glucose concentration. In conclusion, the NPSP in combination with a smartphone application can effectively monitor pH and glucose concentrations in wounds (Videos S8, S9, Supporting Information).

## 3 Conclusion

We have developed an innovative therapeutic approach by incorporating an optical sensor into the NPWT system using 3D printing technology. The NPSP displays color and fluorescence signals measurable via smartphone, enabling real‐time tracking of wound pH and glucose levels during NPWT without necessitating dressing changes. Upon detection of early infection signs through pH changes, we can rapidly administer the drug‐loaded microspheres through the NPSP, concurrently with ongoing NPWT. Comprehensive in vitro and in vivo studies have demonstrated that the NPSP system facilitates continuous wound monitoring during NPWT, offering significant advantages in clearing infections and ameliorating local inflammation significantly as compared to NPWT methods. Looking ahead, we aim to deploy the NPSP system in more complex wound scenarios, enhancing detection and drug distribution precision.

## 4 Experimental Section

### Materials

CCK8 Assay Kit, Calcitonin/PI Cell Viability/Toxicity Assay Kit, Lactate Dehydrogenase (LDH), and L929 Cells (Cat. No. CCL‐1). GOx and HRP were purchased from Sigma‐Aldrich. AWE was purchased and prepared according to established protocols from the literature.^[^ ^70^ ^]^ Transparent resin and black resin were purchased from Hangzhou Nexus Technology Co. Chitosan and vancomycin was purchased from Aladdin Biochemistry. Male Sprague‐Dawley (SD) rats (6–8 weeks old) and male New Zealand White rabbits (3–5 months old) were purchased from Suzhou Jingwei Yu Biotechnology Co. Negative pressure units were supplied by Suzhou Aide Co., Ltd. and Wuhan VSD, China.

### 3D Printed Negative Pressure Patch Device Fabrication, Preparation of Glucose Sensor, pH Sensor, and Vancomycin‐Loaded Microspheres

*Fabrication of 3D Printed Negative Pressure Patch Device*: The process involved creating a design using Shaper3D and converting it to an STL file. The STL file was then imported into the 3D printer (SimpNeed P300). The glucose channel was printed using black epoxy, while the pH‐responsive channel, drug delivery channel, and drainage channel were printed using clear epoxy. The reaction channels were then cleaned, dried, and cured.

*Preparation of Glucose Sensor*: GOx at a concentration of 1 × 10^−9^ m and HRP at a concentration of 200 × 10^−9^ m were added to 2% sodium alginate to mix the solution thoroughly, and the resulting solution was homogeneously dripped into 2% CaCl_2_ solution via a micropump to obtain the glucose sensor. A 20 G injection needle with a flow rate of 1 mL min^−1^, and a tip to CaCl_2_ solution surface height of 10 cm was set in the experiments. The fully cross‐linked glucose sensor microspheres were pressed into molds to obtain a circular glucose sensor with a diameter of 3 mm.

*Preparation of pH Sensor*: The alginate microspheres were obtained by dropping 2% sodium alginate solution into 0.2 mol L^−1^ CaCl_2_ solution at a uniform rate using a high‐pressure electrostatic device. The experiments were conducted using an injection needle (30 G, 1 mL h^−1^, and 10 cm) at a voltage of 8.2 kV. Phenol red was used as a pH indicator. Alginate microspheres were immersed in PBS containing phenol red to form a pH sensor.

*Preparation* *of Vancomycin* *‐* *Loaded Microspheres*: Chitosan has been widely applied to the medical field due to its non‐toxicity, odorlessness, non‐irritating nature, excellent safety and biocompatibility.^[^ ^71^, ^72^ ^]^ That is why chitosan microspheres were chosen for drug loading. First, 1% chitosan solution was dropped uniformly into liquid nitrogen using a high voltage electrostatic device. The injection needle had a pitch of 20 G, a voltage of 8 kV, a flow rate of 0.2 mL min^−1^, and a tip to liquid nitrogen surface height of 17 cm. The resulting spherical chitosan ice beads were dried in a vacuum freeze dryer for 12 h, then crosslinked with 2.5% glutaraldehyde vapor at 20 °C for 12 h, and finally washed with ethanol and ultrapure water to obtain the CSM. V/CSM was produced by immersing CSM in vancomycin solution at varying concentrations at 4 °C for 12 h, followed by drying in a vacuum freeze dryer for 12 h. Five chitosan microsphere samples were prepared according to the following compositions: 1) chitosan microspheres (CSM); 2) chitosan microspheres prepared with 1 mg mL^−1^ of vancomycin solution (denoted as V/CSM1); 3) V/CSM2; 4) V/CSM3; and 5) V/CSM4. The negative pressure sponge was perforated and the holes were cured with PDMS. Meanwhile, the drug delivery device was placed above the holes of the negative pressure sponge to achieve negative pressure drug delivery.

### In vitro Determination of Glucose Concentration and pH

*Glucose Concentration Tests*: For glucose testing, negative pressure sponges were prepared to absorb different concentrations of glucose solutions configured from PBS and AWE. At the same time, the glucose sensor was placed into the glucose reaction channel, dropped into 10 µL of 0.5 × 10^−3^ m H_2_DCF substrate solution, and sealed with 0.5 mm thick quartz glass. The sealed NPSP was placed into the NPWT system and connected to a vacuum pump. In this way, the glucose solution flowed into the glucose reaction channel under negative pressure and came into contact with the glucose sensor. After 30 min, the sample was photographed under UV light (365 nm). Subsequently, the fluorescence intensity of the hydrogel samples were detected at 485 nm excitation light wavelength and 528 nm emission light wavelength using a microplate reader to analyze the quantitative relationship between fluorescence intensity and glucose concentration.

To investigate the effect of red blood cells on the performance of the glucose sensor, a uniform glucose concentration was maintained in all samples. Given that red blood cells make up ≈40% of the blood volume, the samples were divided into six different groups, each with a different concentration of red blood cells: 40%, 20%, 10%, 5%, 1%, and a control group without red blood cells. The glucose hydrogel was immersed in a liquid medium and then the substrate solution was added. The reaction was carried out for 30 min, after which the excess liquid was aspirated, and the fluorescence intensity was measured with a microplate reader.

*pH Tests*: pH was monitored by utilizing a negative pressure sponge to absorb pH (5.5 to 8) solutions configured from PBS and AWE, respectively. While the NPSP system was in operation, pH sensor microspheres were injected using a syringe for pH detection, and photographs of the NPSP were captured. Solutions with varying pH values were added dropwise to pH sensor microspheres. Subsequently, the samples were analyzed spectroscopically using microplate reader to determine their absorption values at 550 nm.

*Smartphone Monitoring of pH and Glucose Concentration*: In this work, images were captured using a portable smartphone, and subsequently processed by ImageJ and MATLAB software to obtain the corresponding functional relationships and formulas. To develop the mobile application, code was written using programming software, and further details can be found in Figure S6 (Supporting Information).

### Characterization of Drug‐Loaded Microspheres, pH Sensors, and Glucose Sensors

*SEM Characterization*: The surface morphology of five chitosan microsphere samples (CSM, V/CSM1, V/CSM2, V/CSM3, V/CSM4), pH sensors, and glucose sensors was characterized using a scanning electron microscope (SEM, Hitachi SU1 510).

### Zone of Inhibition, Antimicrobial Activity Test, and In Vitro Release of Vancomycin

ZOI refers to a circular area surrounding the antibiotic area, in which bacterial colonies cannot grow. The zoneZOI was determined through a paper diffusion method. Briefly, the bacterial suspension was spread evenly over the length of the agar plate, then left at room temperature for 3–5 min. Five milligrams of chitosan microsphere samples (CSM, V/CSM1, V/CSM2, V/CSM3, V/CSM4) were then added into 500 µL of PBS for 4 h. After release, 10 µL of the drug‐released solution was added onto a drug‐sensitive paper. After the paper was air‐dried, the drug‐sensitive paper was clamped with sterile tweezers and placed in the center of the plate. Measurement of the diameter of ZOI was carried out after overnight incubation. In the antimicrobial activity section, 5 mg of chitosan microsphere samples (CSM, V/CSMP1, V/CSMP2, V/CSMP3, V/CSMP4) and 0.5 mL of bacterial suspension were added to 2 mL Eppendorf tubes, and mixed at 37 °C for 4 h. Referring to the previous literature,^[^ ^73^ ^]^ the Rap is calculated using the following equation:

(1) $$\mathrm{Rap}\left(\%\right)=\frac{A-B}{A}\times 100$$

A and B represent the number of live bacteria in the control (CSM) and experimental (V/CSM) groups, respectively.

Ten milligrams of V/CSM1, V/CSM2, V/CSM3, and V/CSM4 were added into 10 mL of PBS and the absorbance was measured at 280 nm by a microplate reader at different time intervals from the solution.

### Biocompatibility of Materials

Hemolysis assay was analyzed by observing and measuring the release of hemoglobin from red blood cells. 2% (v/v) red blood cells were prepared according to previous experimental methods.^[^ ^74^ ^]^ 1 g of pH sensor microspheres was added to PBS free of blood cells to eliminate interference with Phenolsulfonphthalein color. After incubation and centrifugation, OD values were measured. pH sensor OD values were obtained by subtracting the background absorbance from the pH sensor absorbance. The hemolysis rate was calculated using the equation:

(2) $$\mathrm{Hemolysis}\;\mathrm{rate}\left(\%\right)=\frac{OD_{\mathrm{sample}}}{OD_{100\%\;\mathrm{hemolysis}\;\mathrm{group}}}\times 100\%$$

Cytotoxicity of the material was determined using Cell Counting Kit‐8 (CCK8), based on the methodology of an earlier study. Cell viability was calculated using the equation:

(3) $$\mathrm{Cell}\;\mathrm{viability}\left(\%\right)=\frac{OD_{1}-OD_{2}}{OD_{3}-OD_{2}}\times 100\%$$

1 represents the experimental group, 2 represents the blank group, and 3 represents the control group.

To further observe the growth morphology of L929 cells, the cells cultured in conditioned supplemented with NPSP subassembly extract were immuno‐stained. Cell morphology was observed using a confocal light microscope, and cell fluorescence imaging photographs were taken.

### In Vivo Experiments

**Figure 11:**
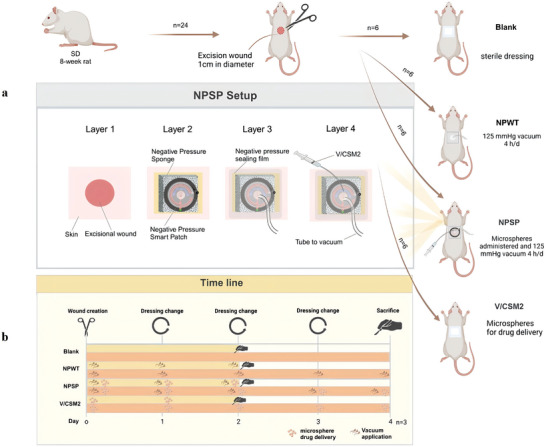
Flow chart of animal experiment. a) Grouping of SD rats and NPSP are shown at different levels of the setup; b) Timeline depicting the time points of wound formation, device application, drug exchange, and rat sacrificing.

*In Vvo Performance of NPSP Administration on Infected Wounds*: Male SD rats weighing between 200–250 g and aged 6–8 weeks were randomly assigned to groups. Twenty‐four rats were subjected to debridement in preparation for surgery. The rats were first anesthetized with 2% to 4% isoflurane vapor in the induction chamber. Subsequently, a circular wound of 1 cm in diameter was surgically created on the dorsum of each rat. After the wound model was established, the rats were inoculated with 0.5 mL of 10^8^ CFU mL^−1^ *S. aureus* suspension. The experimental groups were as follows: six rats were covered with a closed dressing (as a blank group); another six rats were treated with NPWT by applying −125 mmHg pressure for 4 h a day for 4 days (NPWT group). In the third group, six rats were injected with 5 mg of V/CSM2 (the minimum amount of biocide determined in the in vitro experiments) via the NPSP, and then subjected to −125 mmHg for 4 h per day for 4 consecutive days (NPSP group); A final group of six rats received a simple administration of 5 mg of V/CSM2 in the absence of negative pressure. The experimental procedure is shown in **Figure** **11**, and the operating parameters of the negative pressure pump are shown in Figure S7 (Supporting Information).

*Sample Preparation*: Three animals from each of the four groups were sacrificed on days 2 and 4 after wound formation. The wound samples were collected from the center of the wound bed. Three biopsy samples were excised from each study group at each time point. Rapid fixation was performed in 2.5% glutaraldehyde and stored at 4 °C overnight.

*Bacterial Quantification of Infected Tissues*: Three animals in each of the four groups were sacrificed on days 2 and 4 after wound formation, respectively. The wound samples were collected from rats under sterile conditions, subsequently immersed in 1 mL sterile PBS, and placed on ice. The samples were ground with a tissue grinder. The ground tissue solution was diluted and spread evenly on agar plates. The plates were then incubated for 24 h to allow for measurement.

*Immunofluorescence and H&E Staining*: Paraffin sections were processed and stained with primary and secondary antibodies. Subsequently, the nuclei were restrained with 4',6‐diamidino‐2‐phenylindole. Autofluorescence was quenched by the sections were sealed, and the sections were placed under a scanner for image acquisition or under a fluorescence microscope for photographing. Sections were stained using an H&E staining kit.

*Scanning Electron Microscopy Sample Preparation*: Tissues were fixed overnight with 2.5% glutaraldehyde, followed by gradient dehydration and subsequent lyophilization.

*In Vivo Detection of Glucose and pH Concentration*: Two male New Zealand Large White rabbits weighing 3000–3500 g and aged 3–5 months were randomly divided into two groups: the NPWT group and the NPhenolsulfonphthalein group. Vapor anesthesia was induced with 2–4% isoflurane in an induction chamber. A complete 3 cm × 10 cm wound was formed on the back of each rabbit. After establishing the wound model, the rabbits were inoculated with 2.5 mL of 10^8^ CFU mL^−1^ *S. aureus*. Intervention was performed after 30 min. Rabbit wounds in both the NPWT group and NPSP group were treated as with treating the rat wounds described above, except that the microsphere dosage in NPSP group was adjusted from 5 mg to 25 mg. Two groups were treated for four consecutive days, and NPSP was applied to monitor pH concentrations on days 2, 6, and 10, respectively.

### Statistical Analyses and Plotting

Statistical analyses and graphs were performed using GraphPad 9.0 software. All data are expressed as mean ± standard deviation with a sample size of three. For normally distributed data sets with equal variances, comparisons between multiple groups were performed by one‐way ANOVA tests followed by Tukey post‐hoc tests, and comparisons between two groups were made using independent samples *t*‐tests. In all cases, *p* < 0.05 was considered statistically significant, *p < 0.05, **p < 0.01, ***p < 0.001, ****p < 0.0001.

### Ethical

All the experiments in this study were conducted in accordance with the rules for the use and care of animals at the Jiangsu Institute of Parasitic Disease Control. This study was ethically reviewed by the Experimental Animal Ethics Committee of the Jiangsu Institute for the Control of Parasitic Diseases (No. IACUC‐JIPD‐2023119).

## Conflict of Interest

The authors declare no conflict of interest.

## Supporting information



Supporting Information

Supplemental Video 1

Supplemental Video 2

Supplemental Video 3

Supplemental Video 4

Supplemental Video 5

Supplemental Video 6

Supplemental Video 7

Supplemental Video 8

Supplemental Video 9

## Data Availability

The data that support the findings of this study are available from the corresponding author upon reasonable request.
