# Supplementary material for: Negative Pressure Smart Patch to Sense and Heal the Wound
Source: Adv Sci (Weinh). 2024 Nov 28;12(3):2408077. doi: 10.1002/advs.202408077 (PMC11744653; doi:10.1002/advs.202408077)
Supplement: Supplementary file 1 — Supporting Information [file ADVS-12-2408077-s001.docx]

Supplementary Materials for

**Negative pressure smart patch to sense and heal the wound**


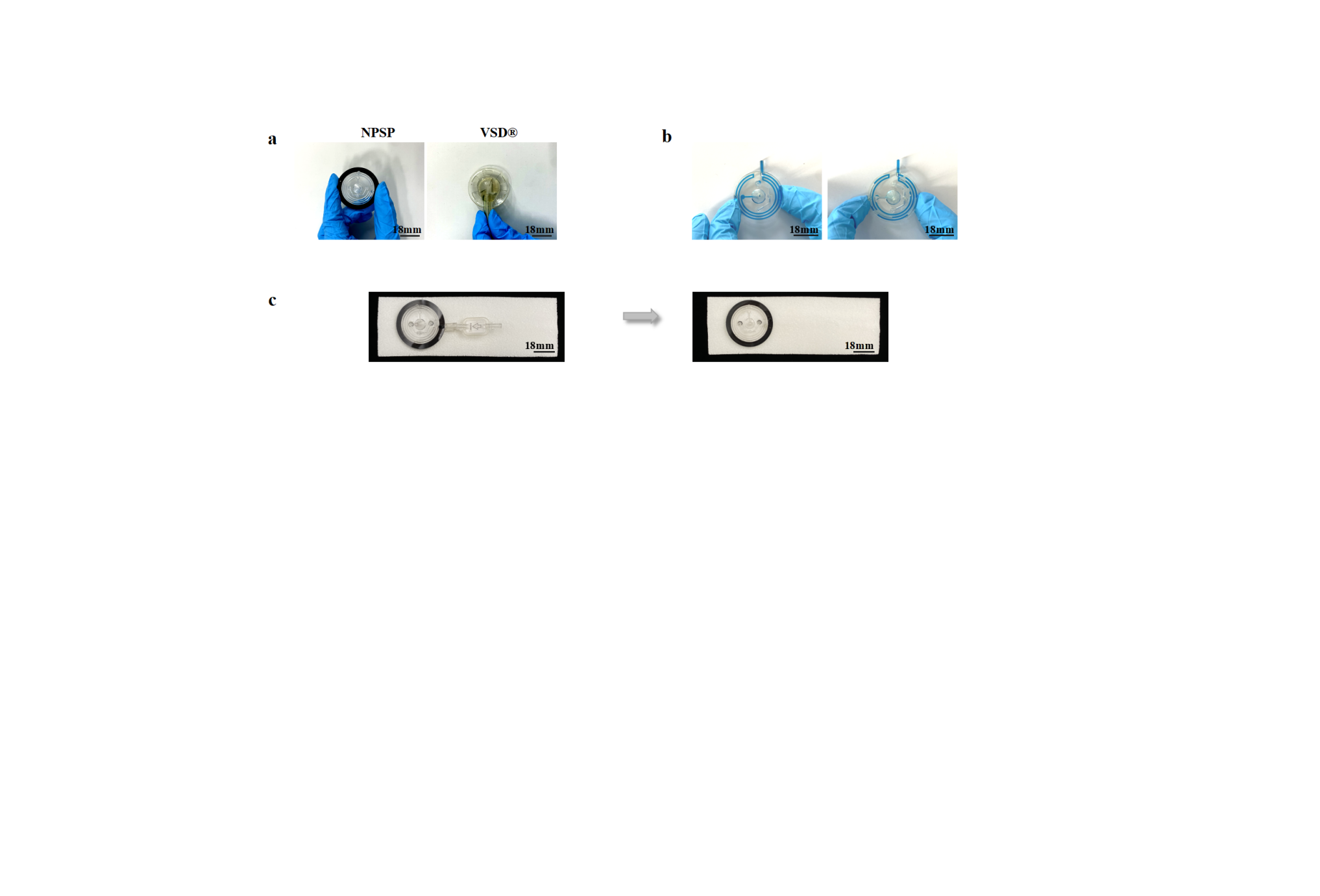


**Figure S1. Comparison of NPSP and Commercial Negative Pressure Fittings and Installation Procedures (a) Photographs of NPSP (left) compared to a commercialized negative pressure patch (VSD^®^) (right); (b) Photographs of dyed water in the pH reaction channel in a laboratory environment with (left) and without (right) a removing filter; (c) Assembly of NPSP and negative pressure sponge.**


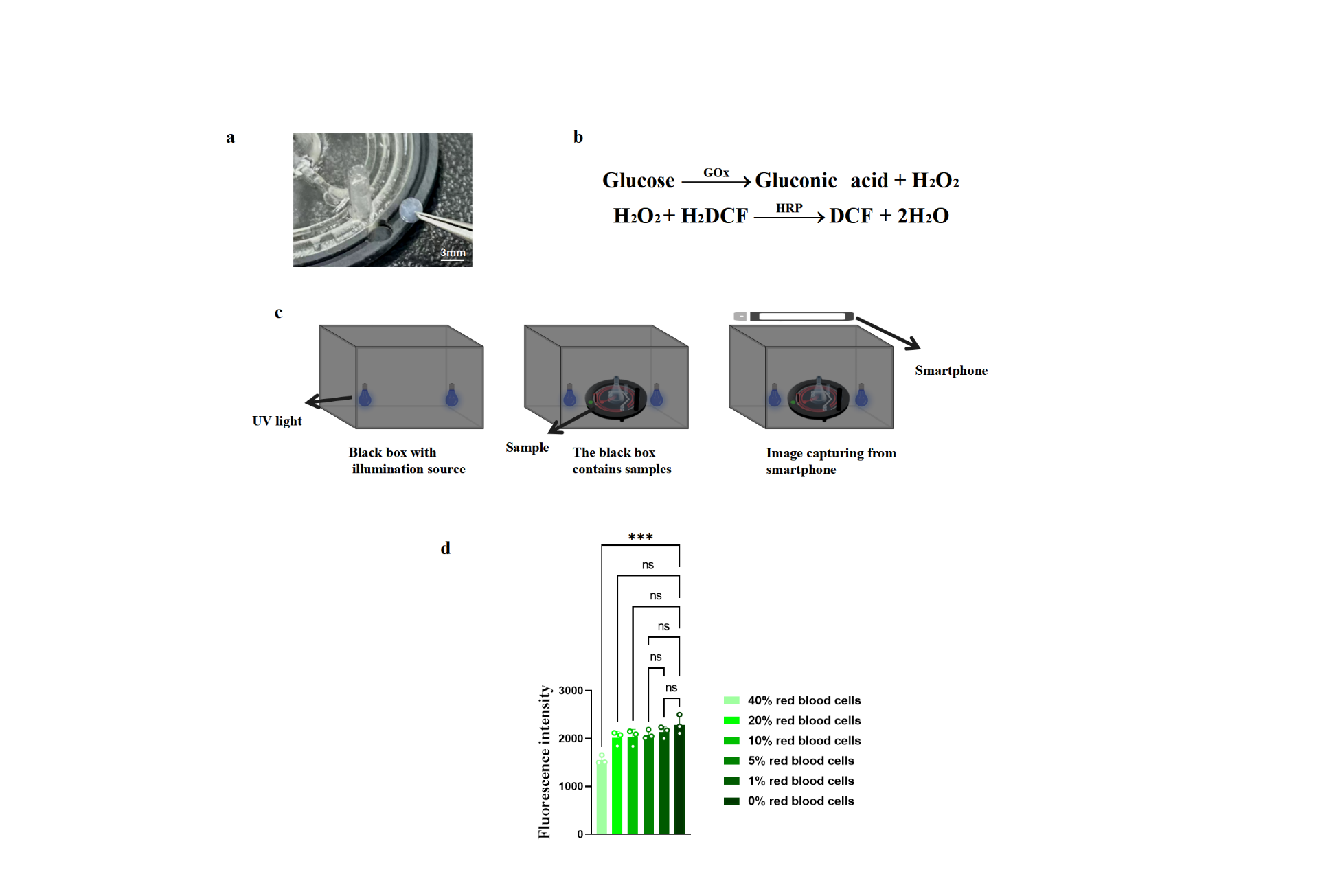


Figure S2. Working principle of glucose sensor and interference study (a) Glucose sensor in the glucose reaction channel; (b) A reaction catalyzed by GOx and HRP; (c) Schematic diagram of the optical path of the glucose sensor; (d) The bar graphs show the fluorescence intensity of the glucose sensor at fixed glucose levels at different concentrations of red blood cells (specifically 40%, 20%, 10%, 5%, 1% and 0%). Results for insets d are expressed as mean ± standard deviation, ***p < 0.001. Statistical analyses were performed using one-way ANOVA followed by Tukey post-hoc test, *n = 3*.


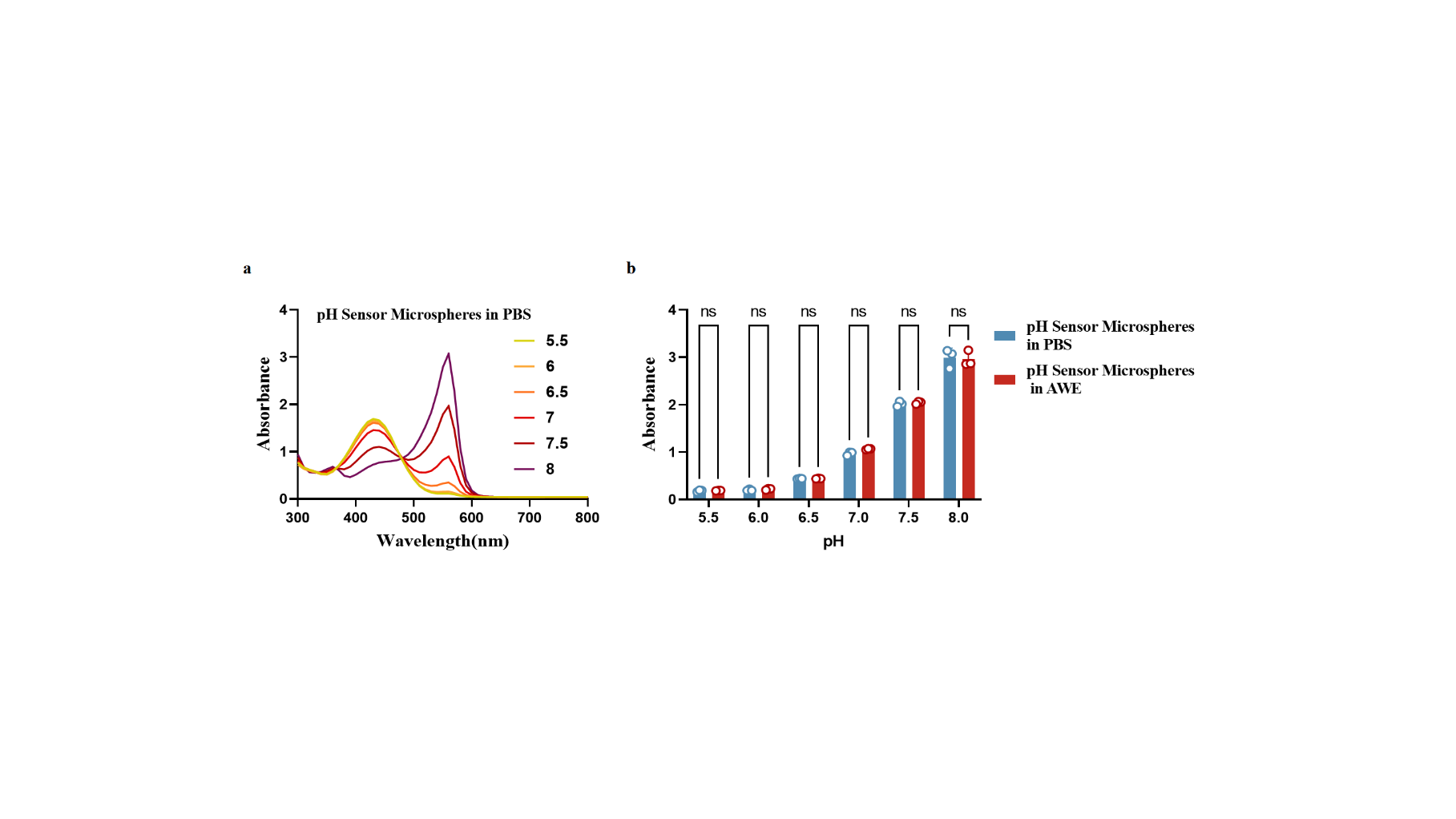


Figure S3. Interference studies of pH sensors (a) Scanning spectra of pH sensor microspheres obtained at various pH values of PBS; (b) Absorption values of pH sensor microspheres measured at 550 nm across varying pH levels. Results in inset b are expressed as mean ± standard deviation, and statistical analyses were performed using the independent samples *t*-test, *n = 3*.


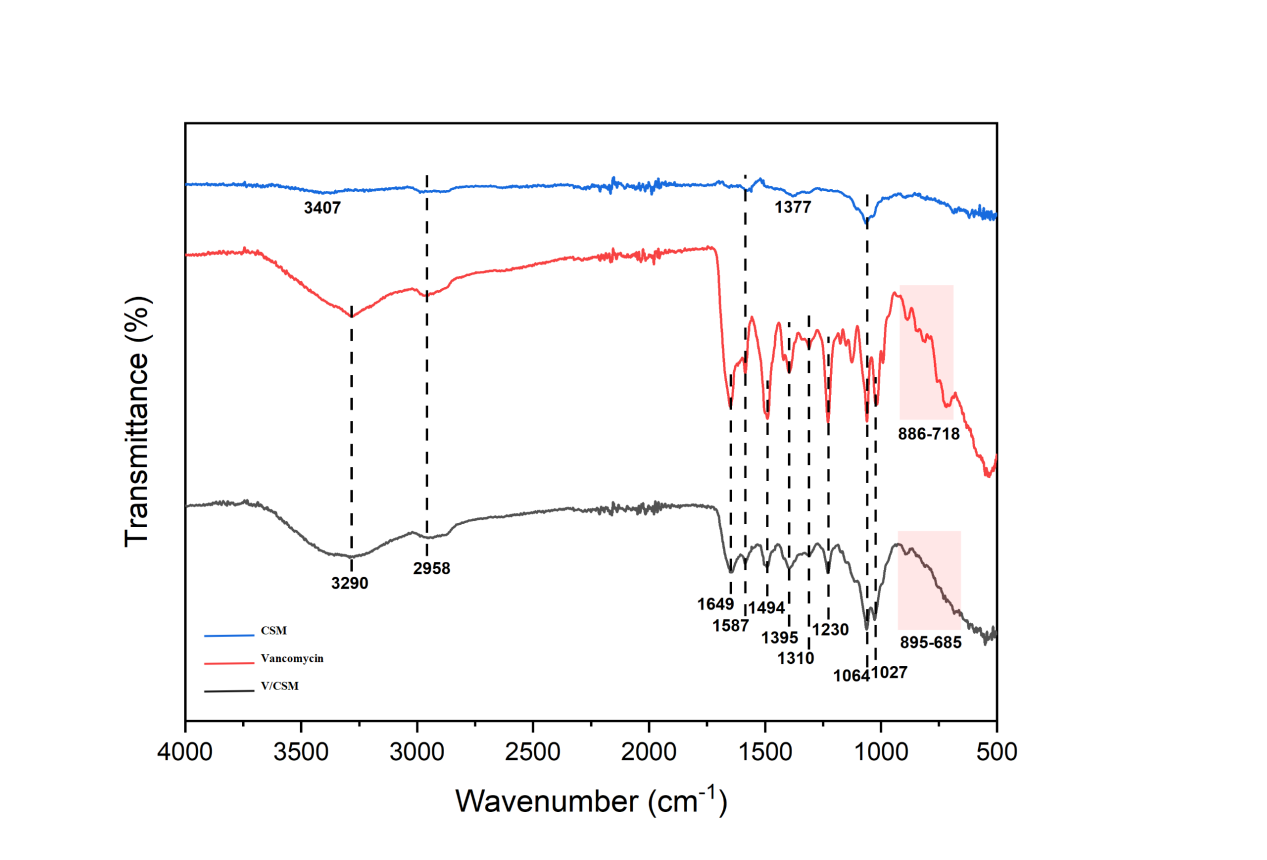


**Figure S4. FTIR spectrum of the CSM, vancomycin, and V/CSM.**

**
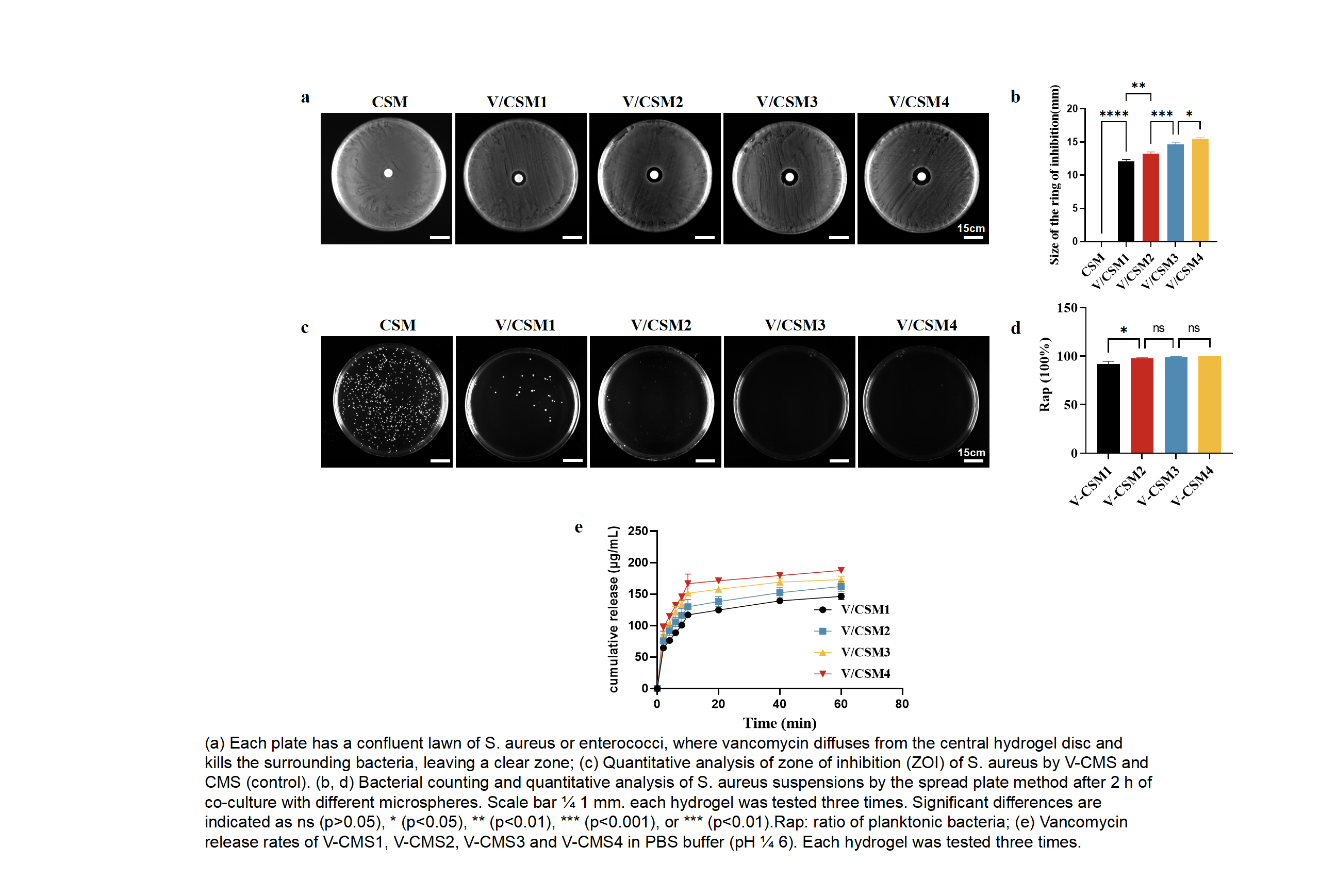
**

**Figure S5. Drug release profile. Vancomycin release rates of V/CSM1, V/CSM2, V/CSM3, and V/CSM4 in PBS buffer (pH = 6). Three tests were performed for each hydrogel.**


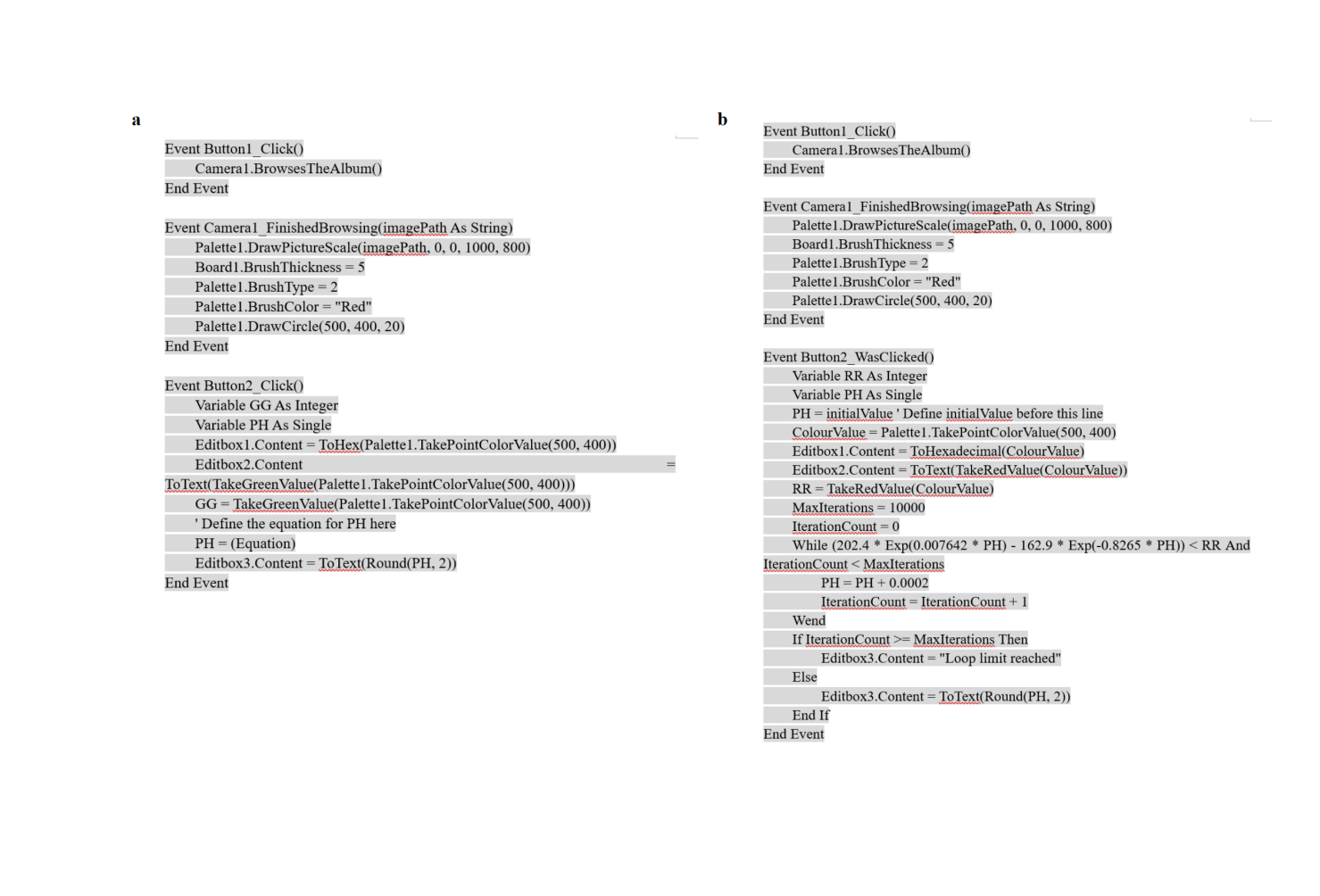


**Figure S6. Code for cell phone programs (a) Program code for pH recognition in smartphones; (b) Program code for recognizing glucose concentration in smartphones.**

**
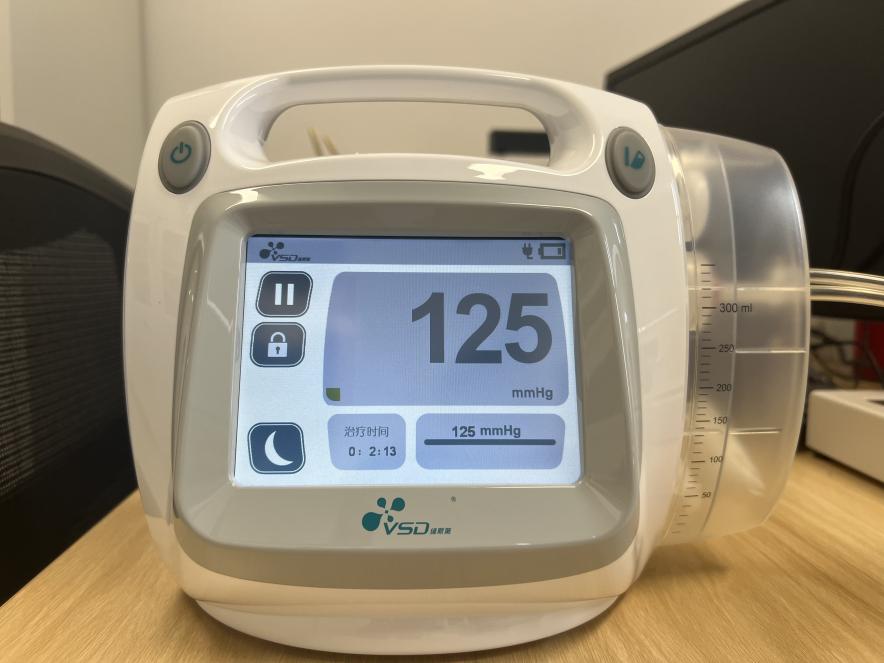
**

**Figure S7. Negative pressure machines used for experiments.**
